# Recent Progress in MXene Hydrogel for Wearable Electronics

**DOI:** 10.3390/bios13050495

**Published:** 2023-04-22

**Authors:** Yi Ren, Qi He, Tongyi Xu, Weiguan Zhang, Zhengchun Peng, Bo Meng

**Affiliations:** 1 Key Laboratory of Optoelectronic Devices and Systems of Ministry of Education and Guangdong Province, School of Physics and Optoelectronic Engineering, Shenzhen University, Shenzhen 518060, China; 2 Guangdong Laboratory of Artificial Intelligence and Digital Economy (SZ), Shenzhen 518107, China

**Keywords:** hydrogel, MXene, wearable sensors, flexible electronics

## Abstract

Recently, hydrogels have attracted great attention because of their unique properties, including stretchability, self-adhesion, transparency, and biocompatibility. They can transmit electrical signals for potential applications in flexible electronics, human–machine interfaces, sensors, actuators, et al. MXene, a newly emerged two-dimensional (2D) nanomaterial, is an ideal candidate for wearable sensors, benefitting from its surface’s negatively charged hydrophilic nature, biocompatibility, high specific surface area, facile functionalization, and high metallic conductivity. However, stability has been a limiting factor for MXene-based applications, and fabricating MXene into hydrogels has been proven to significantly improve their stability. The unique and complex gel structure and gelation mechanism of MXene hydrogels require intensive research and engineering at nanoscale. Although the application of MXene-based composites in sensors has been widely studied, the preparation methods and applications of MXene-based hydrogels in wearable electronics is relatively rare. Thus, in order to facilitate the effective evolution of MXene hydrogel sensors, the design strategies, preparation methods, and applications of MXene hydrogels for flexible and wearable electronics are comprehensively discussed and summarized in this work.

## 1. Introduction

As society and technology flourish, artificial intelligence and smart sensing come to the forefront and show a boom in development at an impressive rate [1,2,3]. The increasing ageing of the population and changes in lifestyle habits have led to more and more people suffering from chronic diseases such as cardiac arrest, asthma, diabetes, and obesity. Therefore, smart wearable sensors for monitoring physical signals (including heart rate, body temperature, blood pressure, etc.) and early disease diagnosis are urgently needed [4,5,6,7,8]. Usually, sensors are designed to work by converting external stimuli (biological, chemical, mechanical, electrical, optical, and thermal) into detectable signals. They require high sensitivity, excellent mechanical flexibility, wide detection range, and stable responsive sensing capability [9,10]. In this regard, hydrogels as an inherently stretchable class of ionic conductors with remarkable mechanical and electrical properties, as well as being biocompatible with human tissues, have been widely developed and used in flexible electronics and robotics for biomedical applications.

**Figure 1:**
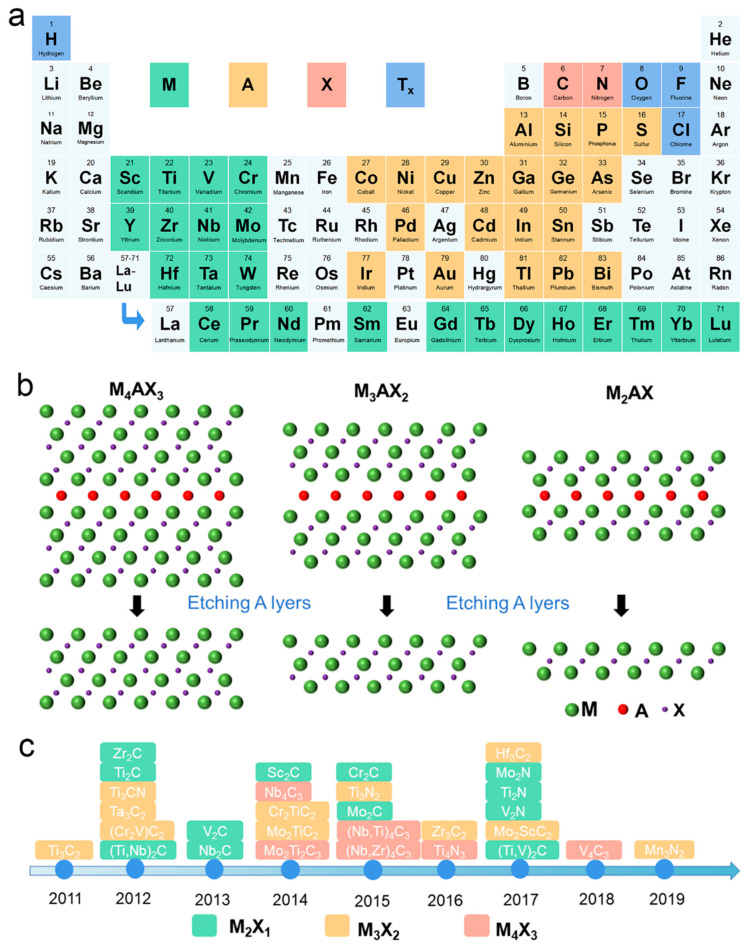
(**a**) Periodic table with MAX phases and MXene compositions. (**b**) The schematics of etching MAX phases as the precursor of three typical MXenes, including M_2_X, M_3_X_2_, and M_4_X_3_ MXene. (**c**) Timeline of progress in MXene synthesis.

As new 2D materials, MXenes were first derived in 2011 from their parent MAX phases (M_n+1_AX_n_) [11], where M refers to an early transition-metal element (e.g., Ti, Zr, V, Nb, Ta, or Mo), A is a group of 13–16 elements, and X is carbon or nitrogen, where n = 1, 2, 3 (Figure 1a) [12,13]. MXenes can be obtained by selectively removing the A layer from the MAX phase since the bonding force of the M-X bond is stronger than that of the M-A bond (Figure 1b). The chemical formula of MXenes is M_n+1_X_n_T_x_, where T_x_ represents surface functional groups, such as F, Cl, or O. Three atomic structures of the MXene family, M_2_X, M_3_X_2_, and M_4_X_3_, have been discovered (Figure 1c), offering great possibilities and feasibility to explore and utilize diverse materials [14].

The rich chemical composition, metallic conductivity, and solution processability of MXene make it a potential material in a wide range of application areas from catalysis, biomedicine, flexible electronics, and energy storage [12,14,15,16,17,18,19,20]. Unfortunately, MXene is susceptible to self-aggregation in aqueous media through van der Waals attraction and hydrogen bonding that limits its potential applications [21,22]. Compared with other 2D materials such as black phosphorus (BP), MXenes exhibit better mechanical strength, electrical conductivity, and hydrophilicity [23,24]. In this regard, when MXenes are incorporated into hydrogel systems, they can offer more possibilities to be engineered into MXene-based soft materials with tunable properties according to specific application requirements [25,26]. However, the preparation of homogeneous MXene-based hydrogels still remains a challenge since MXene nanosheets (NSs) may cluster during the hydrogel preparation [27,28]. Although recent reviews present detailed reports on the construction of various MXene 3D-based composites, reviews on MXene hydrogels are barely reported. There is still a deficiency in collecting a wide range of MXene hydrogel preparation methods and applications. Therefore, we summarize previous methodologies in the field of MXene-based composite hydrogel fabrication, highlight their functional properties, and discuss their potential applications.

## 2. Synthesis Strategies for 2D MXene

So far, MXene has been scaled up with great success in multiple applications, and therefore, the study of the synthesis method of MXene has become a hot research topic. Typically, the two main types of etching methods are top-down and bottom-up approaches, for example, etching from MAX and non-etching approaches (chemical vapour deposition; CVD) [29,30,31]. Typically, a variety of etchants (e.g., HF, HCl-LiF, NaOH, and tetrabutylammonium hydroxide (TBAOH)) have been employed to etch the MAX phase [11,32,33,34]. Meanwhile, many emerging non-etching approaches are booming, such as intercalation and hydrothermal, which can also be used for MXene fabrication. The conditions (such as temperature, etching time, and agitation) for synthetic MXene substantially affect the natural mechanical and chemical behaviour of the terminal product, thus having a direct impact on the performance of MXene-based electronics [35,36]. Hence, this work summarizes the unique approaches for the fabrication of MXene as shown below, and a more fundamental mechanistic investigation of their synthesis is imperative in the expectation that more MXene-based devices can be effectively developed in subsequent studies.

(a) Hydrofluoric acid etching of MAX

**Figure 2:**
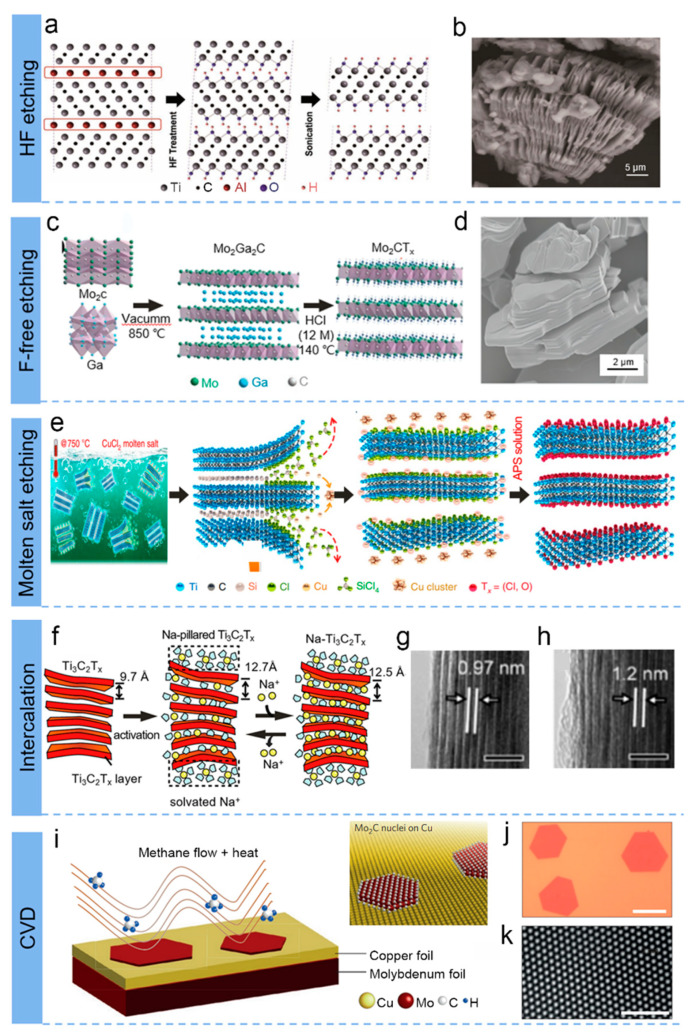
(**a**) Schematic of the exfoliation process for Ti_3_AlC_2_ by HF etching. (**b**) SEM image after HF treatment. (**a**,**b**) Reproduced with permission [11]. (**c**) Schematic illustration of the preparation procedure for fluoride-free Mo_2_CT_x_. (**d**) SEM image for Mo_2_CT_x_ MXenes. (**c**,**d**) Reproduced with permission [41]. (**e**) Schematic fabrication of Ti_3_AlC_2_ by immersion in CuCl_2_ Lewis molten salt. Reproduced with permission [42]. (**f**) The mechanism of Na^+^ intercalation into MXene layer. (**g**,**h**) The TEM pictures of MXene after sodiation and desodiation. (**f**–**h**) Reproduced with permission [43]. (**i**) MXene Mo_2_C produced by chemical vapour deposition. (**j**) Optical and (**k**) high-resolution TEM images of Mo_2_C crystal. Reproduced with permission [44].

Selective etching has been extensively utilized for the preparation of MXene since the successful etching of Al layers in the MAX phase of precursors (Ti_3_C_2_) using concentrated hydrofluoric acid (HF) was first reported in 2011 [11]. HF is mainly employed for selective etching of the a-element layer present in the MAX precursor, notably for the synthetic carbide-based MXenes [14,37]. It is believed that the concentration and synthesis time of HF solution are the key effects and parameters for controlling the size, morphology, and surface properties of MXene synthesis [38,39]. As shown in Figure 2a, the powder of the MAX phase precursor is stirred in an aqueous solution of HF at a specific concentration, and subsequently, the solid is separated from the supernatant by filtering the mixture of the stirred solution. As a result, a loose accordion-like structure (Figure 2b) is formed, and the etching reaction is given below [40]:

Ti_3_AlC_2_(s) + HF(l) → Ti_3_C_2_T_x_(s) + AlF_3_(s) + H_2_(g) (1)

The MXene obtained from this process is usually abbreviated as MXT_x_, where T_x_ stands for the F, O, and OH end groups, respectively.

(b) Fluoride-free etching

HF solution etching is considered the standard preparation method for MXene. However, the usage of HF etchant is intensely toxic and corrosive, requiring long etching times in the conventional synthesis route, which greatly hinders the deeper investigation of MXene. For safety reasons, researchers have been searching for alternative etchants to replace HF [45,46]. A mixture of hydrochloric acid (HCl) and lithium fluoride (LiF) has been reported by Ghidiu et al. as a safer etchant [41]. Apart from the enhanced safety, the etchant also ensures the peeling of multilayers through the insertion of Li^+^ and also its hydration capsule into the internodal interstitial interval. In addition, both the etching and intercalation were conducted simultaneously, and varying the properties and composition of the fluoride salt at the time of intercalation yields many MXenes with tunable structures and properties [32,47]. Song’s group demonstrated a fluoride-free strategy to fabricate Mo_2_C MXenes with high efficiency of about 98%, as displayed in Figure 2c [41]. The as-obtained Mo_2_C, shown separately platelet-like structure (Figure 2d), different from other MXenes (Ti_3_C_2_T_x_ and V_2_CT_x_) etched by HCl [48,49]. Due to the selected surface functional groups created by the HCl etching process, the as-produced Mo_2_C electrodes offer high electrochemical capabilities when applied in batteries and supercapacitors.

(c) Molten salt etching of MAX

The MAX phases utilized for the preparation of MXene are predominantly those of the MAX phase (where A = Al element), and they account for approximately 20% of the ternary MAX phase only [50]. Therefore, it is crucial to develop new techniques for etching the other 80% of the MAX phase family members into MXene materials [51,52]. The synthetic MXene by Lewis acid melt salt method, as a flourishing strategy [53,54], has greatly enriched the MXene species.

Huang et al. first reported a redox-controlled A-site to fabricate Ti_3_C_2_ by the etching of MAX (Ti_3_SiC_2_) phases in CuCl_2_ Lewis acidic melts at 750 °C, as exhibited in Figure 2e. In addition, the variety of MAX precursors (A-site element: Al, Zn, Ga) also proved the feasibility of this method. Talapin et al. proposed a class of Zn-based MAX phases and Cl-terminated MXene via various molten inorganic salts in 2017 [52]. The synthetic route consists of two main steps. Firstly, new MAX phases (Ti_3_ZnC_2_, Ti_2_ZnC, Ti_2_ZnN, and V_2_ZnC) were synthesized according to the substitution reactions [55,56,57]. Afterwards, Cl-terminated MXene (such as Ti_3_C_2_Cl_2_ and Ti_2_CCl_2_) were successfully fabricated due to the strong Lewis acidity of molten ZnCl_2_, which benefited from the subsequent exfoliation conducted in the special reaction environment of excess molten ZnCl_2_. The reaction formulations are as below [58]:

Zn + 2HCl → ZnCl_2_ + H_2_ ↑ (2)

Ti_3_AlC_2_ + 1.5ZnCl_2_ + 0.5Zn + AlCl_3_ ↑ (3)

Similar to the reaction in the molten salt of ZnCl_2_, a variety of MXene were successfully prepared by adjusting the chemical composition of MAX precursors and the Lewis acid melt type. Currently, researchers reported the successful etching of different MAX phase precursors (where A = Al, Si, Ga, Ge, In, and Sn) [36,59]. 

In addition, Lewis acid systems comprising CdCl_2_, FeCl_2_, CoCl_2_, CuCl_2_, AgCl, and NiCl_2_ have been successfully employed to greatly broaden the variety of MXene [42,60,61]. Additionally, surface termination conversion in molten inorganic salts has been demonstrated to be another valid strategy to produce more MXene. For example, dispersing Ti_3_C_2_Br_2_ in molten CsBr/KBr/LiBr and adding Li_2_Te or Li_2_S, respectively, Ti_3_C_2_Te and Ti_3_C_2_S can be achieved [62]. Similarly, the variety of T_x_-terminated MXene can be enriched with this generally feasible technique, which now extends to halogens (-Cl, -Br, -I), halogens (-S, -Se, -Te), and bare MXene [59,63].

(d) Intercalation methods of MAX

In many cases, it is always a necessary step to intercalate the layered nanomaterials from bulk into 2D, which owns lots of unique properties [64,65,66]. The obtained MXenes by wet etching are generally multilayered materials, whereas monolayered and few-layered MXenes demonstrate unique chemical and physical properties and have a wider range of applications [67,68,69,70]. Hence, the production of high-quality and stable multilayer or monolayer MXene is crucial. Intercalation and delamination are essential steps in obtaining MXenes with few or single layers, which are typically performed sequentially or simultaneously in the etching process. At present, typical intercalating agents, which can be inserted between MXene layers to destroy or weaken the van der Waals bonds between layers, are mainly divided into three categories: molecular, cationic, and organic bases. 

Gotosi et al. first reported the intercalation of multilayers Ti_3_C_2_ by using hydrazine and co-intercalation with N,N-dimethylformamide (DMF) as the molecule-mediated intercalator, resulting in an increase of c-lattice parameters (from 19.5 to 26.8 Å) [71]. In addition, other intercalating agents, such as urea, hydrazine hydrate (HM), HM dissolved in DMF, and dimethyl sulfoxide (DMSO), were also precisely able to delaminate the multilayered MXenes into monolayers. 

Currently, cation-mediated intercalation (with various cations, including NH^4+^, H^+^, Li^+^, Na^+^, K^+^, Ca^2+^, Mg^2+^, and Al^3+^) is also a common strategy for the preparation of few-layer or monolayer MXene [58,72,73,74]. Yamada et al. revealed the mechanism of reversible Na+ intercalation/deintercalation into the interlayer space of MXene Ti_3_C_2_T_x_ in a nonaqueous Na+ electrolyte via electrochemical reaction, as shown in Figure 2f [43]. At the onset of sodization, dissolution intercalation of Na^+^ and solvent molecules occurred between Ti_3_C_2_T_x_ layers, which caused the Ti_3_C_2_T_x_ interlayer distance to swell from 0.97 to 1.2 nm (Figure 2g,h).

In general, molecules and ionic compounds used as intercalants are not feasible for all MXenes, while organic macromolecules are available. For example, Ti_3_C_2_T_x_ could be intercalated and delaminated with DMSO, whereas Ti_3_CNT_x_ could not, as reported by Naguib et al. [68]. The organic-based intercalants mainly consist of polar organic molecules, including urea, propylene carbonate (PC), isopropylamine (i-PrA), N, N-dimethylacetamide (DMAc), cetyltrimethylammonium bromide (CTAB), tetrabutylammonium hydroxide (TBAOH), and tetramethylammonium hydroxide (TMAOH) [75,76,77,78]. So far, Ti_2_CT_x_, Ti_3_C_2_T_x_, V_2_CT_x_, Mo_2_CT_x_, Mo_1.33_CT_x_, TiVCT_x_, Ti_3_CNT_x_, and Ti_4_N_3_T_x_ have been intercalated and delaminated successfully by TBAOH [79,80,81,82] and Ti_3_C_2_T_x_, Nb_2_CT_x_, and Nb_4_C_3_T_x_ by i-PrA [81,83,84].

Although the liquid-phase intercalation method can produce MXenes with large lateral size, it is difficult to obtain large-area, stable, and thin-layer MXenes with excellent performance. Thus, this technique still needs to be further investigated in future MXene research.

(e) Direct chemical vapour deposition of MXene

In contrast to the generally wet etching methods mentioned above, bottom-up synthesis methods, including chemical vapour deposition (CVD), are also available for the synthesis of MXenes. Cheng’s group fabricated the high-quality, ultrathin, and large-scale (over 100 µm in size) MXene of Mo_2_C by CVD by using methane as the carbon source and a Cu foil sitting on a Mo foil as the substrate, as shown in Figure 2i [29,44]. The results of optical image (Figure 2j) and high-angle annular dark-field-scanning transmission electron microscopy (HAADF-STEM) revealed defect-free hexagonal α-Mo_2_C crystals (Figure 2k), which are thicker than one lattice unit, making them robust and chemically stable. This versatile synthetic method can be extended to other carbides [85,86].

Till now, the synthesis of monolayered MXene by CVD has not yet been reported and is subject to further development. Compared to wet chemical etching, MXene fabricated via the CVD method has fewer defects, higher purity concentration, and better density uniformity. Thus, large lateral-dimensioned MXene can be obtained to study the inherent physical and chemical properties. Moreover, CVD is a new way to prepare different kinds of MXene. However, high manufacturing costs and safety issues limit the large-scale fabrication of MXene by CVD, and bottom-up synthesis methods are highly demanded to be developed. The CVD-grown materials possess the advantage of large lateral dimensions and few defects, which facilitates the study of their intrinsic properties and provides a new way to prepare different kinds of MXene. However, high manufacturing costs and safety issues limit the large-scale commercialization of CVD, so there is a need to develop more bottom-up synthesis methods.

## 3. Fabrication of MXene-Based Hydrogels

MXene NSs inevitably tend to polymerize and re-stack due to the strong van der Waals interlayer attraction, thus rendering MXene difficult to form hydrogels on its own [24,26]. However, MXene has abundant surface terminations, excellent hydrophilicity, and extensive plasticity, which makes it susceptible to combine with other materials to yield a variety of hybrids. As a result, the addition of another component (i.e., cross-linker) to the hydrogel matrix to balance the hydrophilicity of MXenes and maintain the 3D assembly of 2D NSs is often critical in the preparation of MXene-based hydrogels. Furthermore, MXenes inevitably interact with oxygen in the environment, which limits their practical use. Physical and chemical surface engineering techniques have been reported to protect MXenes from oxidation [34]. During the formation of MXene hydrogels, the high electronegativity and low work function of the atoms on the MXene surface allow organic ligands to deprotonate and bind to the MXene surface. Thus, oxidation of MXene can be avoided [24,87,88]. The MXene-based hydrogels can be classified into three categories and are presented as follows: (1) inorganic-material-MXene nanocomposite hydrogels, (2) polymer-MXene nanocomposite hydrogels, and (3) metal-MXene hybrid nanocomposite hydrogels.

### 3.1. Inorganic Material-Assisted MXene Nanocomposite Hydrogels

The limited accessible crosslinking sites on the surface of MXene restricted its functional activity, while graphene oxide (GO) as a 2D carbon-based material possesses good conductivity and high specific surface area, as well as mechanical properties, allowing the formation of interactions with the MXene NS surface as a gelling agent [89]. Hence, the self-stacking phenomenon between MXene NSs was greatly reduced, and the role of MXene in hydrogel was enhanced.

**Figure 3:**
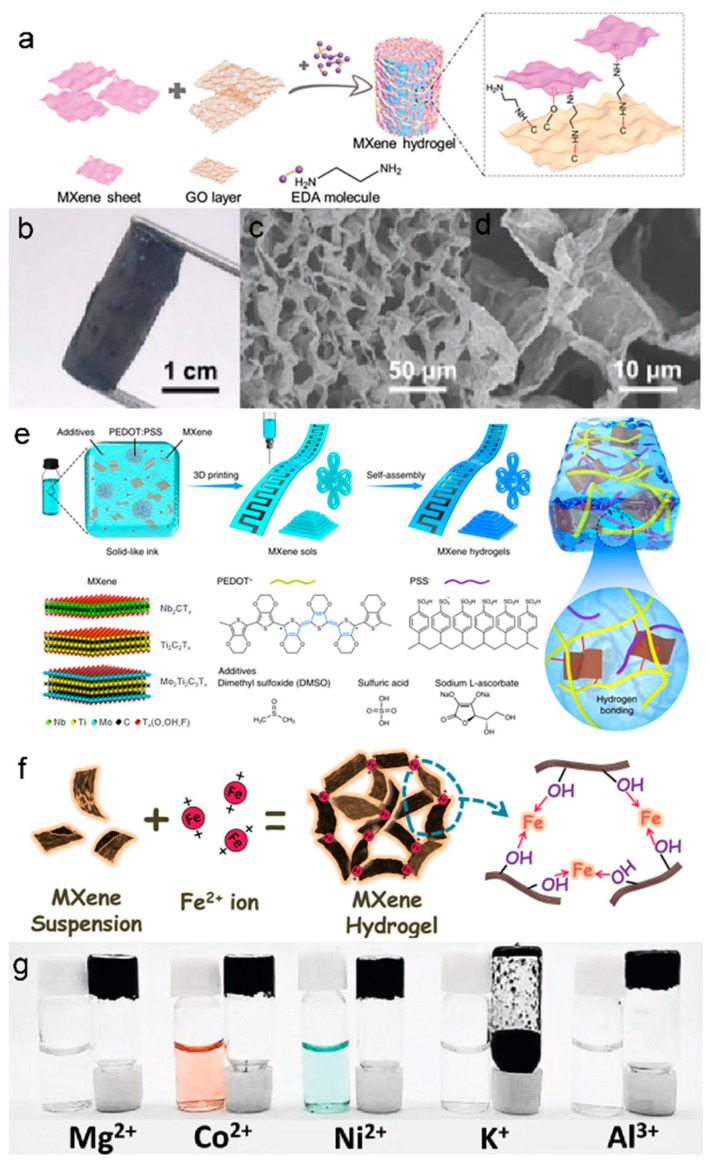
(**a**) Schematic illustration of the formation process of MXene/GO/EDA hydrogel. (**b**) Optical photographs and (**c**,**d**) SEM images of MXene hydrogel at different magnifications. (**a**–**d**) Reproduced with permission [22]. (**e**) The process of 4D printing of MXene hydrogels. Reproduced with permission [87]. (**f**) The schematic of metal-ion-initiated interaction of MXene NSs. (**g**) The optical photo of MXene mixed with different ions. (**f**,**g**) Reproduced with permission [88].

Xu et al. first reported rGO/MXene 3D macroscopic hydrogel via an organic-free self-convergence process. In this process, graphene oxide was converted to reduced graphene oxide (rGO) by using Ti_3_C_2_T_x_ as a reducing agent under mild conditions, resulting in the removal of some hydrophilic oxygen-containing groups and the enhancement of the hydrophobic and π-conjugated structure of rGO, thus enabling the assembly of the rGO/MXene 3D skeleton [89]. Shang’s group proposed 3D MXene-based hydrogels assembled with GO and ethylenediamine (EDA) by a self-assembly method [22]. As the reduction of GO is induced by Ti_3_C_2_T_x_, EDA promotes the formation of oxygen suspension bonds by opening the epoxy rings on the GO flakes. As shown in Figure 3a, Ti_3_C_2_T_x_ was then attached to these dangling bonds to form MXene-rGO hetero structures, which were transformed into hydrogels by van der Waals forces between layers of heterogeneous NSs. Figure 3 b-d display the well-defined and interconnected 3D porous network of MXene-based hydrogel, ranging from the submicron level to several microns.

### 3.2. Polymer-Assisted MXene Nanocomposite Hydrogels

The incorporation of MXene into polymer hydrogel networks has recently attracted considerable attention since this network can be extensively swollen by water, which gives them outstanding versatility in electronic devices [24]. Combining MXenes with other polymers (polyvinyl alcohol (PVA), poly(3,4-ethylenedioxythiophene): poly (styrene sulfonate) (PEDOT: PSS), polyvinylpyrrolidone, cellulose, chitosan, and others) [25,87,90,91,92,93], using MXene surface groups to interact with the polymers forms hydrogels by polymerization reactions. Generally, interactions between MXene NSs and other polymers in the hydrogel network result from the intertwining of polymer chains, as well as ionic interactions, hydrogen, and/or covalent bonds. 

Nicolosi et al. presented a facile 4D printing technique for manufacturing MXene-based hydrogels consisting of MXene, PEDOT: PSS, and additives (DMSO, H_2_SO_4_, and sodium L-ascorbate), and such a technique has been extended to the MXene family, including Nb_2_CT_x_, Ti_3_C_2_T_x_, and Mo_2_Ti_2_C_3_T_x_ [87]. As displayed in Figure 3e, the composite inks were firstly made into various patterns by 3D printing and subsequently transformed from MXene sols into MXene hydrogels by self-assembly. The obtained 4D-MXene hydrogels demonstrated superior specific capacitance (232.9 F g^−1^ at 10 V s^−1^) and enabled low-temperature operation (–20 °C). Wang et al. prepared a flexible and stretchable TENG by using MXene NSs and PVA hydrogel encapsulated as electrodes and utilized it in wearable self-powered sensors for body motion monitoring [25].

### 3.3. Metal-MXene Hybrid Nanocomposite Hydrogels

During the gelation process described above, nonetheless, the inevitable oxidation led to partial degradation of the properties of the formed MXene-based hydrogels [24,94,95]. Therefore, to alleviate the effects of oxidation and accelerate the phase separation of MXenes from water, a faster gelation process is required to significantly suppress the restacking of MXene NSs [96]. Ye et al. proposed a metal-assisted electrogelation method to directly generate MXene hydrogels with porous structures and tunable characteristics [97]. This controllable strategy provides more sophisticated patterning with greater complexities and efficiencies than 3D printing or laser patterning, which involves multi-step operations or has large device dependency. During the electro-gelation process, the released metal cations are initiated by electrolysis in which electrostatic interactions occur between the cations and the MXene NSs. The rapid gelation of MXene in aqueous dispersions initiated by divalent metal ions (Fe^2+^) was first reported by Yang et al. The strong interactions between metal ions and the -OH groups on the surface of the MXene surface played a crucial role [88]. The gelation process, illustrated in Figure 3f, employs metal ions (Fe^2+^) as the linking points to enhance the construction of MXene NSs into a three-dimensional network due to their strong binding energy with the -OH groups on the MXene surface. The other metal ions (Mg^2+^, Co^2+^, Ni^2+^, and Al^3+^) also confirmed the successful synthesis of the metal-MXene-hybrid-hydrogel system via the metal-ion-mediated gelation method (Figure 3g).

## 4. MXene-Based Hydrogels for Wearable Sensors

As a soft and stretchable material, hydrogels can respond to a wide range of chemical and physical stimuli by inducing measurable changes in geometric, optical, and electrical properties, and thus they have been widely used in wearable electronics. Nevertheless, the sensitivity of most conventional hydrogel sensors is rather low under mechanical stimuli (strain/pressure), and they often suffer from signal hysteresis and fluctuations due to their viscoelastic properties [98]. Benefiting from their excellent electrical conductivity, mechanical deformability, and diverse surface functional groups, MXene materials play an important role in making conductive hydrogels [99,100,101]. For instance, MXene can interact with the hydrogel network to enhance its mechanical and electrochemical properties, and thus, hydrogel’s sensing properties and biocompatibility can be improved, correspondingly. Hence, MXene-based hydrogels are expected to be utilized in wearable electronic devices, such as electronic skin, smart sensors, and personalized healthcare monitoring instruments.

### 4.1. MXene-Based Hydrogels for Pressure Sensors

Typically, pressure sensors can be categorized into piezoresistive, capacitive, piezoelectric, and triboelectric pressure sensors depending on different signal transformations [102,103,104,105,106,107,108]. Among them, piezoresistive sensors are the core components of various wearable electronic devices due to excellent temperature stability, high sensitivity, and short response time. Generally, the metallic conductivity and the relative sliding layers give MXenes great adjustable resistance range, which are highly regarded in pressure-sensitive materials with excellent application prospects.

**Figure 4:**
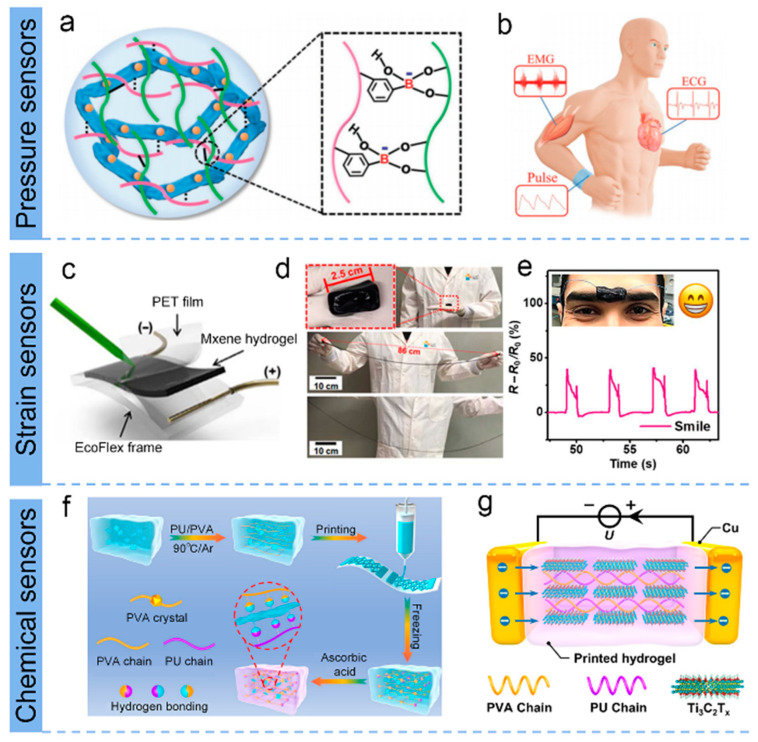
(**a**) Schematic of the MXene hydrogel and its applications in (**b**) human healthcare monitoring. (**a**,**b**) Reproduced with permission [99]. (**c**) Schematic for signature sensing. (**d**) Photographs demonstrating the stretchability of MXene-based hydrogel. (**e**) Resistance changes of MXene-based hydrogel in response to facial expressions of a smile. (**c**–**e**) Reproduced with permission [100]. (**f**) The direct-ink-writing (DIW) printing process of MXenes bonded with polyurethane/polyvinyl alcohol hydrogel. (**g**) Schematic illustration of temperature sensing. (**f**,**g**) Reproduced with permission [101].

Conductive hydrogel flexible sensors have been extensively utilized for personalized medicine and e-skin, but it remains a great challenge to simultaneously obtain highly sensitive sensing (especially electrophysiological signals) for wearable human–computer interactions while accelerating wound healing for aftercare. Thus, a healable, degradable, and antibacterial epidermal sensor was fabricated by Wan et al. for detecting weak physiological signals and simulating effective treatment of wound infections [99]. As shown in Figure 4a, the obtained MXene-based hydrogel was fabricated by adding Ag NPs/MXene into the polymer network (composed of guar gum (GG) and phenylboronic acid grafted sodium alginate (Alg-PBA)) [99]. This MXene-based hydrogel forms a multifunctional epidermal sensor that can sensitively monitor a wide range of energetic activity and small electrophysiological signals, providing important clinical information for rehabilitative training and cardiovascular-related diseases (Figure 4b).

### 4.2. MXene-Based Hydrogels for Strain Sensors

The addition of MXene to hydrogels offers a distinct possibility to improve the performance of hydrogel strain sensors by combining the merits of both MXene and hydrogel, which exhibits promising electronic properties and scalable mechanical flexibility [109,110,111]. 

Figure 4c–e exhibited the MXene-based hydrogel (consisting of MXene (Ti_3_C_2_T_x_), PVA, water, and anti-dehydration additives) prepared by Alshareef et al. exhibited excellent tensile strain sensitivity with a gauge factor (GF) of 25, tenfold higher than that of the original hydrogel [100]. In addition, the hydrogel has extremely high tensile properties, self-healing, and good integration and adhesion to a variety of surfaces, making it promising in applications for touch sensing and biosignal monitoring. Yu’s group developed a strain-sensitive MXene-based hydrogel (MNH) using ethylene glycol (EG) as the dispersion medium instead of water [23]. The MNH possesses excellent freeze resistance (−40 °C) and durable moisture retention stability (8 d) compared to conventional hydrogels. Additionally, this MNH could be assigned as a flexible sensor that monitors biological movements of the human body at very low temperatures, featuring a relatively wide strain range (up to 350% strain) and a high measurement factor (44.85). Recently, most MXene-based stretchable electronics use MXenes as conductive nanofillers, while Alshareef et al. employed Ti_3_C_2_T_x_ MXene acting as a multifunctional cross-binding agent that activates various hydrogels and rapid gelation, starting from various monomer or polymer precursors (e.g., PAA-, PAM-, PDMA-, PNIPAM-, PHEMA-, PANI-, and PEGDA-MXene hydrogels) [112].

### 4.3. MXene-Based Hydrogels for Chemical Sensors

The need of innovative diagnostic methods for human health is continuously growing, and user-friendly biosensors have gained considerable research interest. Personal diagnostic sensing devices are capable of monitoring patients and healthy individuals for premature diagnoses and early prevention of diseases [113,114,115]. So far, a variety of hydrogel-based biosensors have been successfully developed for simultaneous detection of different biomarkers or physiological signals via electrical and optical transducing strategies (i.e., diabetes-related glucose [116,117], cardiovascular disease-related triglycerides (TG), and heartbeat (pulse)) [118,119]. MXene materials are excellent candidates for the exploitation of new electrochemical sensing and biosensing devices due to their unique surface chemistry, high electrical conductivity, and biocompatibility, showing their great potential for future portable and wearable health monitoring and diagnostic tool sets [120,121].

Temperature, humidity and air pollution are the essential factors affecting human life. Sulphur dioxide (SO_2_) is a typical gaseous pollutant that is extremely harmful to human health and the ecosystem, which can cause significant breathing and cardiovascular diseases in humans. Additionally, Wang’s group reported a MXene/TiO_2_/SnSe sensor driven by a triboelectric nanogenerators (TENG), consisting of a vinyl chloride trifluoroethylene (ECTFE) film and an ionic hydrogel electrode [122]. The prepared sensor has an excellent response (ΔU/Ua = 170% at 30 ppm) for SO_2_ gas detection, which is 14 times greater than that of the resistive sensor. Lin and co-workers fabricated a Ti_3_C_2_/sodium alginate (SA) hybrid hydrogel with high performance of biochemical detection at tissue interfaces (sensitivity of detection of hydrogen peroxide: 600 nA μM^−1^ cm^−2^; LOD: 12 nM) [123].

The versatility of flexible sensing systems for hydrogels remains a challenge in terms of cost, integration difficulty, and device fabrication, hindering specific application scenarios. Therefore, Huang’s team proposed a 3D-printed direct-ink-writing technique with low cost and scenario applicability to successfully prepare MXene-bonded hydrogel sensors with excellent strain and temperature-sensing properties simultaneously [101]. The schematic processes for the direct-ink-writing printing of MXene Ti_3_C_2_T_x_ assembled with polyurethane/polyvinyl alcohol (PU/PVA) hydrogel and the MXene-based hydrogel for temperature sensing are shown in Figure 4 f,g. Such MXene hybrid hydrogel exhibits a GF of 5.7 (0–191% strain) and high temperature sensitivity (TCR of −5.27% °C^−1^ at 0 to 30 °C and −0.84% °C^−1^ at 40 to 80 °C). The synthesis strategies of MXene-based hydrogels and performance of electrical performances of various MXene architectural devices are summarized in Table 1.

## 5. Conclusions and Perspectives 

### 5.1. Conclusions

In recent years, MXene hydrogels have demonstrated great perspectives in wearable sensor applications. However, the fabrication and stability of MXene hydrogels still require in-depth investigation. In this review, the synthesis strategies for 2D MXene and fabrication methods for MXene-based hydrogel have been comprehensively discussed, revealing that the dispersibility of MXene can be improved by modifying the surface of MXene NSs and 2D MXene can be used as a reaction platform to prepare self-assembled hydrogels. By interacting with MXene, the hydrogel demonstrates enhanced mechanical and electrical characteristics comparable to traditional hydrogels, thus resulting better sensing performance. Additionally, MXene-based hydrogels show good biocompatibility that allow them to be used in biomedical applications. Finally, various types of wearable sensors made by MXene-based hydrogels have been introduced, proving great potential in applications for electronic skin, health monitoring, and soft energy-storage apparatus.

### 5.2. Perspectives

The MXene-based hydrogels have the following advantages over hydrogels prepared with other 2D nanomaterials such as graphene: (1) They have high hydrophilicity that promotes good dispersion and stability of MXene-derived photodynamic and photothermal agents in physiological media. (2) The covalent and noncovalent crosslinking of MXene-based hydrogels are relatively weaker, allowing for dynamic assembly and disintegration. (3) MXene-based hydrogels have a variety of preparation methods with environmentally friendly and biocompatible properties, and the tunable surface functionalities can be modified for the desired usage. Although MXene-based hydrogels demonstrate great advantages, there is still a long way to go before their utilization in actual massive practical applications.

## Figures and Tables

**Table 1:** Summary of the synthesis strategies and performance of MXene-based hydrogels.

Hydrogel Composition	Type and Derivative	Synthesis Strategy	Role of MXene	Key Features	Application	Ref.
Ti_3_C_2_/ sodium alginate (SA)	Hydrogel	In situ co-assembled through one-step electro-gelation method	Conductive nanofiller	Conductivity of up to 0.4 S/m; Mechanical strength down to 80 kPa; Excellent electrochemical performance (sensitivity: 600 nA μM^−1^ cm^−2^)	Electrochemical sensing	[123]
MXene/PU/PVA	Hydrogel	Direct-ink-writing by 3D printing	Crosslinker; Conductive nanofiller	Gauge factor (GF) of 5.7 (0–191% strain); Response time of 240 s; Stability over 5000 cycles	Strain and temperature sensing	[101]
MXene/chitosan	Hydrogel	A chitosan-induced self-assembly strategy	Conductive nanofiller	Eminent electroconductivity (4×10^4^ S cm^−1^) and sensitivity (gauge factor of 11); Optimal tensile strength of 190 kPa; Excellent mechanical strength (of up to 1900%) and flexibility	Wearable strain sensors	[91]
MXene/Fe^2+^	Hydrogel	Metal-ion-initiated interaction of MXene	Host materials	Supercapacitor electrode (≈ 226 F g^−1^ at 1 V s^−1^)	Energy storage devices	[88]
MXene	Hydrogel	Universal 4D-printing technology	Self-gelator	3D porous architectures, large specific surface areas, high electrical conductivities, and satisfying mechanical properties	Electrochemical energy storage	[87]
AgNPs/MXene/GG/Alg-PBA	Hydrogel	Dynamic crosslinking	Conductive nanofiller	Degradation of 45 days	Epidermic Sensor	[99]
MXene/PVA	Hydrogel	Chemical crosslinking method	Crosslinking; Conductivity	Stretchable property of about 200%	Self-powered electronic devices	[25]
PVA/SA/MXene	Hydrogel	A green method without using chemical crosslinking agents	Conductive nanofiller	High stretchability of up to 263%; Stability up to 1000 cycles	Strain sensor	[124]

## Data Availability

No applicable.
